# Mental health impacts of nurses caring for patients with COVID-19 in Peru: Fear of contagion, generalized anxiety, and physical-cognitive fatigue

**DOI:** 10.3389/fpsyg.2022.917302

**Published:** 2022-07-25

**Authors:** Lucy Tani Becerra-Medina, Monica Elisa Meneses-La-Riva, María Teresa Ruíz-Ruíz, Aquilina Marcilla-Félix, Josefina Amanda Suyo-Vega, Víctor Hugo Fernández-Bedoya

**Affiliations:** ^1^School of Nursing, Universidad César Vallejo, Lima, Peru; ^2^School of Education, Universidad César Vallejo, Lima, Peru; ^3^School of Management, Universidad César Vallejo, Lima, Peru

**Keywords:** fear, anxiety, fatigue, COVID-19, nurses, health care workers

## Abstract

The health crisis caused by COVID-19 has resulted in the physical and emotional deterioration of health personnel, especially nurses, whose emotional state is affected by the high risk of contagion, the high demands of health services, and the exhausting working hours. The objective of this research was to determine the relationship between fear, anxiety, and fatigue of nurses caring for patients with COVID-19 in a second level public hospital in Peru. This study presents a quantitative approach and correlational level, cross-sectional, and non-experimental design. The sample consisted of 145 nurses who attended patients with COVID-19 in health care areas. The results show a significant relationship between fear of contagion and physical-cognitive fatigue (*p* < 0.001; *r* = 317) and a significant relationship between generalized anxiety and physical-cognitive fatigue (*p* < 0.001; *r* = 480). It is concluded that in this context, both fear of contagion and generalized anxiety are related to physical-cognitive fatigue.

## Introduction

The advent of COVID-19 in the Chinese population in 2019 quickly expanded and became a pandemic, prompting government officials throughout the world to implement severe, even extreme, measures to combat the disease's spread (Gupta et al., 2019; Materassi, 2019). To regulate it, a social and paradigm shift was required, which included the employment of physical social distancing measures; the use of personal protection equipment; and even the worldwide shutdown of economic, educational, and health activities in order to maintain society's health (Valero Cedeño et al., 2020; Caputo et al., 2021; Casaccia et al., 2021; Mendez-Lopez et al., 2022).

With the above, it can be asserted that for this reason, in Peru, health professionals (especially nurses) were added important changes in the scheduling of shifts, such as working days from 6 to 24 h uninterrupted. In addition, there were complaints from hospital staff in which they stated that they were limited in the satisfaction of basic needs such as food and rest, and other situations such as being away from their loved ones, added to high demand from patients and families for quick attention and admission to a bed in the critical care area, as well as limitation and shortage of protective equipment, among others (Cabral, 2022). Similar situation was reported in many other countries (Dutta and Kumar, 2020).

Previous studies has demonstrated that, in addition to posing a major risk to people's lives, epidemic-type illnesses have a detrimental influence on their mental health, eliciting anxiety, depression, and other negative emotions in both health care staff and patients (Liu et al., 2012, 2021; Buckner, 2020; Duncan, 2020; Dutta and Kumar, 2020; Newburn, 2020; Zhang et al., 2020, 2021; Ammar et al., 2021; Cipolotti et al., 2021; Phiri et al., 2021).

Moreover, international research detailed that frontline staff working in hospital centers have experienced depressive symptoms, emotional fatigue, anxiety, and other mental health problems during the COVID-19 epidemic (Cohen and Nica, 2021; Kirkman, 2021; Wells and Miklencicova, 2021).

The COVID-19 pandemic has had a significant and negative impact on the nursing workforce (Chan et al., 2021). The International Council of Nurses reported that by the end of 2021, more than 1,600 nurses worldwide have died from COVID-19 when infected while working on the frontline (CIE, 2020). In this regard, it is important to emphasize that, very particularly, this situation is similar in Peru, a country in which 7,780 nurses were infected with COVID-19 and 90 died in 2021 (Diario Gestión., 2021).

Peru was one of the countries hardest hit by the pandemic in Latin America, with the highest mortality rate of 9 per 100,000 inhabitant in 2021 (Pan American Health Organization., 2021). This situation forced the sector to dictate strategies in terms of health personnel to cope with the high demand; hospital services were prioritized especially in emergency and ICU, leaving the first level of care devoid, services were greatly saturated, coupled with limitations in the provision of protective equipment (Ministerio de Salud del Perú., 2021).

This complex context resulted in the frontline staff perceiving uncertainty and fear during the care process, especially in the hospitalization area where an unexpected and dizzying evolution of the disease is clearly observed, compromising the health and even leading to death of an increasing number of elderly and young people (Chen et al., 2020; Zhang et al., 2020). Finally, it is necessary to assess the impact on mental health related to fear, anxiety, and physical-cognitive fatigue of nurses in times of pandemic in order to assume strategies, improvement plans, and psychological interventions to ensure the quality of work life of nurses working in health institutions (Obando Zegarra et al., 2020; Oliva-Yatlequé and Chávarry-Ysla, 2021; Sánchez-De la Cruz et al., 2021).

## Materials and methods

The research was quantitative, descriptive relational, observational, and cross-sectional (Hernández-Sampieri and Mendoza Torres, 2018). The population was estimated through the online Netquest platform, obtaining a sample of 145 nurses working in the care units for patients with COVID-19 in care areas of national hospitals in Peru; all of them are female. The data were collected in June 2021.

In addition to collecting sociodemographic data, 3 scales were used to measure the variables:

(a) To measure the fear of contagion, the COVID-19 Fear Scale was used. This scale was originally developed by Ahorsu et al. (2020), and subsequently psychometrically validated (Tzur Bitan et al., 2020). Because the nurses in Peru speak Spanish, the scale translated and validated by Sánchez Teruel and Robles Bello (2021) was used (FCV-19S), which was used in Latin American studies (Villalba-Arias et al., 2020), including Peru (Huarcaya-Victoria et al., 2020). It is important to emphasize that a measurement in 2020 reported that this instrument had an optimal level of internal consistency (ω > 0.89 and α > 0.83). This adapted scale consists of seven items that reflect how a person feels, thinks, or acts toward COVID-19. The response options comprise a Likert scale with five alternatives (strongly disagree, disagree, neither agree nor disagree, agree, and strongly agree), while scores range from 7 to 35. The higher the score, the greater the fear of cororonavirus-19.

(b) The Generalized Anxiety Disorder Scale (GAD-7) (Spitzer et al., 2006) was used to measure the generalized anxiety. This instrument consists of seven items with Likert-type responses with a scale of 0 and 21 points, which allows the participant to be diagnosed (0–4 points = no symptoms of anxiety; 5–9 = mild symptoms of anxiety; 10–14 = moderate symptoms of anxiety; and 15–21 = severe symptoms of anxiety). A score of 10 or more defines the presence of anxiety and indicates the need for assessment by a specialized professional (Hinz et al., 2017; Ahn et al., 2019).

c) To measure the physical-cognitive fatigue, the Physical and Cognitive Fatigue Scale of 15 items and Likert-type responses with a scale of 1 to 7 (where 1 = yes, this is totally true; 7 = no, this is not true) was used (Vera et al., 2008). This instrument was validated in Chile in 2008 and assesses how the person perceives physical and cognitive fatigue, obtaining a Cronbach's alpha of 0.85 and 0.78, respectively.

## Results

The sociodemographic information of the respondents is presented in Table 1. According to it, the age group with the highest prevalence is 41 to 50 years (50%). An interesting fact that emerges from this analysis is that no nurses under 30 years of age were identified in the sample. It is likely that the COVID-19 patient care area is staffed by experienced personnel.

**Table 1:** Sociodemographic characteristics of the nursing professional.

**Age**	**Frequency**	**Percentage**
30–40	30	20.0%
41–50	70	50.0%
51–60	43	60.0%
**Job contract**	**Frequency**	**Percentage**
Permanent	21	14.5%
CAS	16	11.0%
CAS-COVID	108	74.5%
**Marital status**	**Frequency**	**Percentage**
Single	60	41.4%
Married	16	11.0%
Cohabitant	12	8.3%
Divorced	54	37.2%
Widowed	3	2.1%
**Housing composition**	**Frequency**	**Percentage**
With cohabitant	73	50.3%
With children	30	20.7%
With parents	19	13.1%
Alone	11	7.6%
More than 1 option	12	8.3%
**Has been infected with COVID-19**	**Frequency**	**Percentage**
Yes	95	65.5%
No	50	34.5%

In terms of employment status, most of the nurses (74.5%) are CAS COVID-19 (a high-paying contract implemented by the government for the exclusive treatment of patients with COVID-19 during the state of emergency); also, 41.4% of the surveyed nurses are single, while 37.2% are widowed. It is likely that precisely this group of people require higher incomes, so they sought to occupy jobs under these conditions.

In terms of housing composition, 50.3% live with a cohabitant. This is quite common in Peru, a country where more and more people prefer to live together before deciding to get married.

Finally, at the date of data collection (June 2021), 65.5% reported not having been infected by COVID-19. Staff working on-site in hospitals are tested for COVID-19 at least once a week, so this data show how well protected nurses who have day-to-day contact with patients infected with the virus are.

Because the study sample was composed of female nurses, despite the interest of the research team, it was not possible to make comparisons of means.

Table 2 shows the relationship between fear of contagion and physical-cognitive fatigue, as it is observed that the sig. (bilateral) is < 0.001. Likewise, the intensity of the relationship is low positive (*r* = 0.317).

**Table 2:** Correlation matrix: fear of contagion and physical-cognitive fatigue.

**Correlations**				
			**Fear of contagion**	**Physical-cognitive fatigue**
Spearman's Rho		Correlation coefficient	1.000	0.317
	**Fear of contagion**	Sig. (2 tailed)	-	<0.001
		N	145	145
		Correlation coefficient	0.317	1.000
	**Physical-cognitive fatigue**	Sig. (2 tailed)	<0.001	-
		N	145	145

Table 3 shows the relationship between generalized anxiety and physical-cognitive fatigue, since it is observed that the sig. (bilateral) is < 0.001. Likewise, the intensity of the relationship is medium positive (*r* = 0.480).

**Table 3:** Correlation matrix: generalized anxiety and physical-cognitive fatigue.

**Correlations**				
			**Generalized anxiety**	**Physical-cognitive fatigue**
Spearman's Rho		Correlation coefficient	1.000	0.480
	**Generalized anxiety**	Sig. (2 tailed)	-	<0.001
		N	145	145
		Correlation coefficient	0.480	1.000
	**Physical-cognitive fatigue**	Sig. (2 tailed)	<0.001	-
		N	145	145

## Discussion and conclusion

COVID-19 is a public health issue that has resulted in a large number of illnesses and fatalities worldwide; nevertheless, despite efforts, there is no effective treatment for this disease. Personal protection measures including social distancing, mask wearing, and frequent hand washing hygiene have been implemented in response to the epidemic. Nursing personnel must implement extraordinary safety precautions in the healthcare sector to ensure a safe atmosphere. There is, however, a high risk associated with (a) interaction with other professionals at work, (b) interaction with the patient with COVID-19, and (c) interaction with their family setting. These three areas are thought to be the most significant causes of nursing professionals' worry, anxiety, and weariness.

According to the findings of this study, the sociodemographic characteristics were that most of the nursing professionals are in the age range of 30–60 years, in the labor condition 73.1% are appointed and marital status are married, working time is 10–20 years, living with husband and children, and 34.5% acquired COVID-19. National and international institutions report a high morbidity and mortality rate in nurses (CIE, 2020; Diario Gestión., 2021).

The results could imply that the mental health of nurses may be seriously compromised by the characteristics of their professional work when faced with a pandemic that despite the time of onset still shows fragility and unspecificity of the evolution and treatment with high demands and exhausting working hours due to the high demand of patients at high risk, in which, although fear of contagion, generalized anxiety, and physical-cognitive fatigue are phenomena that are experienced daily, they have a direct impact on the emotional health of the caregivers (Obando Zegarra et al., 2020; Oliva-Yatlequé and Chávarry-Ysla, 2021; Sánchez-De la Cruz et al., 2021), giving rise to virtual dating (Carrión Degrande Moreira and Ferreira Furegato, 2021).

In relation to fear of contagion and physical-cognitive fatigue, it is positively low. Likewise, health care workers have prevalence to emotional problems during the COVID-19 pandemic, being vulnerable to psychological distress (Villalba-Arias et al., 2020) and fears in nurses are due to high exposure to the COVID-19 virus (Tzur Bitan et al., 2020) since they are exposed to prolonged times due to the shifts they are assigned, lack of personal protective equipment, high work overload, and demand for patient and family care. However, it is important to highlight that despite the fact that nurses are trained to work in risky situations in critical services, this situation was unexpected and accompanied by uncertainties before the pandemic, which increased fears, causing deterioration in the emotional environment (Furman et al., 2020).

In this sense, physical-cognitive fatigue is a problem that directly affects health personnel causing fragility and vulnerability, presenting difficulties to relax, constant worries, feeling nervous, anxious, among others. International studies show that fatigue results from the pressure and work demands to respond to the health problems faced by patients with COVID-19, which, however, despite precarious situations, nurses demonstrate an attitude of commitment to ensure and protect life even though their mental health is greatly affected (Palomino-Oré and Huarcaya-Victoria, 2020). On the other hand, the increase in the number of hours worked and not being able to take sufficient breaks can cause sleep and rest disturbances, elements of vital importance necessary to preserve adequate mental health and work performance (Hinz et al., 2017; Ahn et al., 2019).

In relation to fear of contagion and physical-cognitive fatigue, it is medium positive. These statements indicate the emotional impact constantly experienced by nurses when assuming responsibility for giving nursing care for the recovery of the patient with COVID-19 (Bedoya Jojoa, 2020).

Finally, given the risk of contagion, the human losses of health professionals, family members, and patients have had an emotional impact, causing alterations in mental health, which are manifested in various patterns of behavior, requiring the support of professionals specialized in mental health, to mitigate the impact that directly affects the care and their daily interactions.

## Data availability statement

The raw data supporting the conclusions of this article will be made available by the authors, without undue reservation.

## Ethics statement

The studies involving human participants were reviewed and approved by Comité de Ética de la Universidad César Vallejo. The patients/participants provided their written informed consent to participate in this study.

## Author contributions

All authors listed have equally contributed to the work and approved it for publication.

## Funding

This study was carried out and funded by the Universidad César Vallejo, within the framework of the work plan outlined in RVI N° 052-2019-VI-UCV.

## Conflict of interest

The authors declare that the research was conducted in the absence of any commercial or financial relationships that could be construed as a potential conflict of interest.

## Publisher's note

All claims expressed in this article are solely those of the authors and do not necessarily represent those of their affiliated organizations, or those of the publisher, the editors and the reviewers. Any product that may be evaluated in this article, or claim that may be made by its manufacturer, is not guaranteed or endorsed by the publisher.
